# An archaeal RNA binding protein, FAU-1, is a novel ribonuclease related to rRNA stability in *Pyrococcus* and *Thermococcus*

**DOI:** 10.1038/s41598-017-13062-3

**Published:** 2017-10-04

**Authors:** Yoshiki Ikeda, Yasuhiro Okada, Asako Sato, Tamotsu Kanai, Masaru Tomita, Haruyuki Atomi, Akio Kanai

**Affiliations:** 1 Institute for Advanced Biosciences, Keio University, Tsuruoka, 997-0017 Japan; 2 Graduate School of Engineering, Kyoto University, Kyoto, 615-8510 Japan

## Abstract

Ribosome biogenesis and turnover are processes necessary for cell viability and proliferation, and many kinds of proteins are known to regulate these processes. However, many questions still remain, especially in the Archaea. Generally, several ribonucleases are required to process precursor rRNAs to their mature forms, and to degrade rRNAs for quality control. Here, we found that FAU-1, which is known to be an RNA binding protein, possesses an RNase activity against precursor 5S rRNA derived from *P. furiosus* and *T. kodakarensis* in the order Thermococcales *in vitro*. An *in vitro* analysis revealed that UA sequences in the upstream of 5S rRNA were preferentially degraded by addition of FAU-1. Moreover, a *fau-1* gene deletion mutant of *T. kodakarensis* showed a delay of exponential phase, reduction of maximum cell number and significant changes in the nucleotide sequence lengths of its 5S, 16S, and 23S rRNAs in early exponential phase. Our results suggest that FAU-1 is a potential RNase involved in rRNA stability through maturation and/or degradation processes.

## Introduction

FAU-1 (*P. furiosus* AU binding 1) is a protein that was first identified in *Pyrococcus furiosus*, a member of the order Thermococcales in the Euryarchaea. The protein is highly conserved in Thermococcales (shown in Fig. S1) and is speculated to be a potential orthologue of *E. coli* ribonuclease E (RNase E) which is one of the enzymes responsible for ribosomal RNA (rRNA) processing. FAU-1 has been characterized as an RNA-binding protein that binds to AU-rich sequences^1^. The amino acid similarity between FAU-1 and the N-terminal region of RNase E is approximately 25%^1^, indicating very low similarity. Furthermore, the catalytically active center of the RNase E/G domain in FAU-1 was not predicted by the protein families database (Pfam, http://pfam.xfam.org)^2^; however, the S1 domain and a partial region of the RNase E/G domain were predicted in FAU-1 (Fig. S1). The S1 domain is an RNA-binding region and the partial ribonuclease E/G domain corresponds to the 5′ sensor region involved in the recognition of the RNA substrates of RNase E^3,4^. However, it has not yet been demonstrated whether FAU-1 possesses RNase activity or not.

All organisms have developed quality control systems for the regulation of rRNAs, in which ribonucleases play integral roles. rRNA is initially transcribed as a precursor rRNA that undergoes multiple processing steps, including cleavage by various RNases, to generate mature rRNAs. RNases also participate in rRNA quality control by rapidly degrading aberrant rRNAs^5–7^, which is a well-studied process, particularly in bacteria. It has been reported that the RNase E/G family proteins are among the most important RNases responsible for rRNA maturation and quality control^8–12^. RNase E recognizes the 5′ end of single-stranded RNA and has endoribonuclease activity^13,14^. In *Escherichia coli*, RNase E is involved in the processing of precursor 5S rRNA and 16S rRNA, and is also associated with the stability of mature 16S rRNA and 23S rRNA^9,15,16^. RNase E requires either Mg^2+^ or Mn^2+^ ions for its efficient RNase activity and depends on a Zn^2+^ ion to form a functional tetramer^3,17–19^.

**Figure 1:**
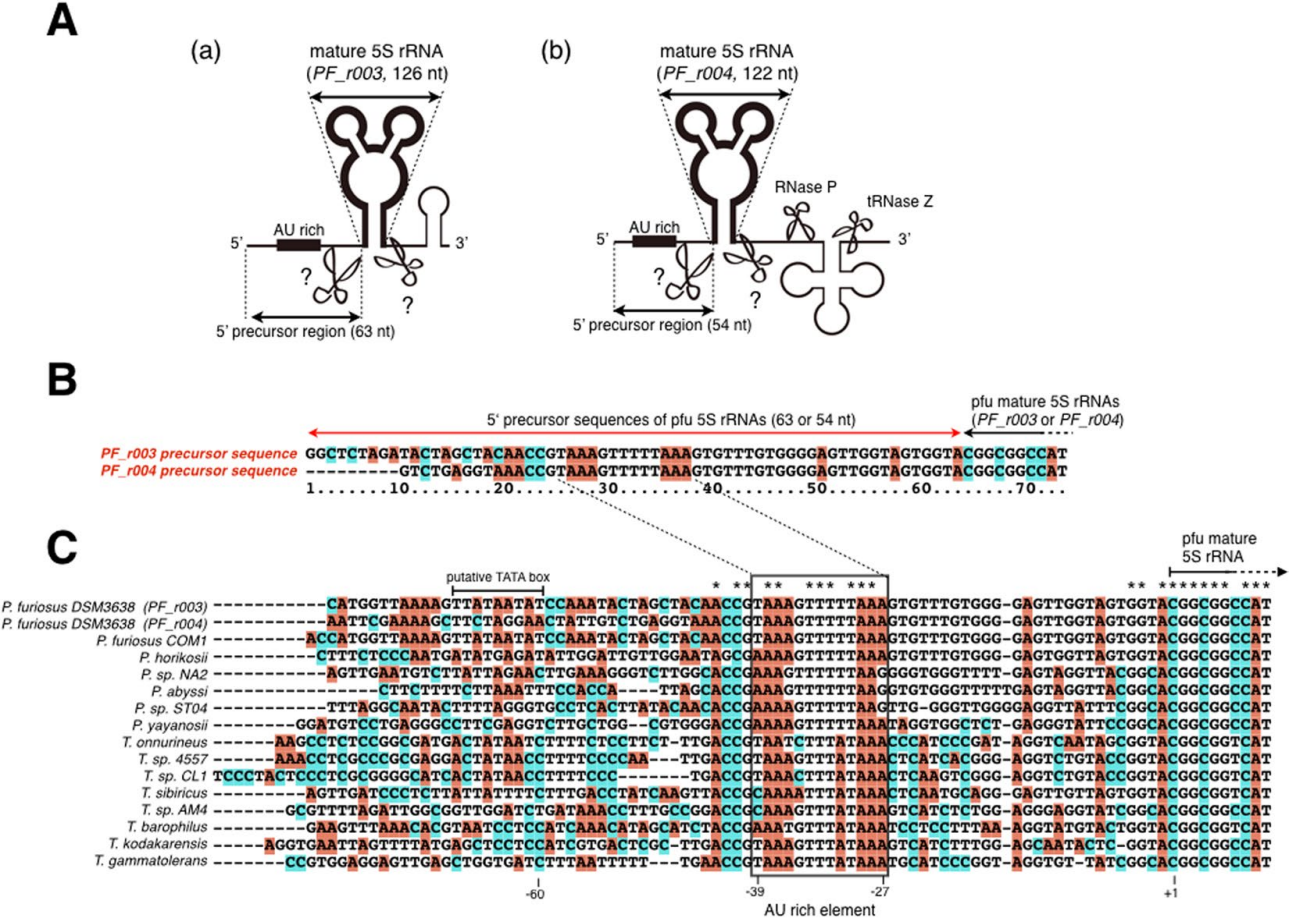
The 5′ precursor sequences of *Pyrococcus furiosus* 5S rRNAs. (**A**) Schematic representations of two pfu 5S rRNA genes (a) *PF_r003* and (b) *PF_r004* and their peripheral regions in the *P. furiosus* genome. The 5′ precursor regions of the pfu 5S rRNAs are shown with double-headed arrows. (**B**) Sequence alignments of the 5′ precursor regions of the 5S rRNAs determined in this study. (**C**) DNA sequence alignments of the regions upstream from the 5S rRNA genes in Thermococcales. Highly conserved AU-rich elements in the 5′ precursor regions of the 5S rRNAs are boxed. The −60 to −68 region is predicted as a putative TATA box according to the UCSC Archaeal Genome Browser database.

In recent studies, several RNases related to rRNA biogenesis have been identified in the Archaea. For example, the Nob1 enzyme is reported to process the 3′ end of the precursor 16S rRNA (pre-16S rRNA) in *Pyrocuccs horikoshii* (Euryarchaea, Thermococcales)^20^. However, the reports of rRNA processing in Archaea are still extremely limited compared with those for the Bacteria. Importantly, Thermococcales consists of hyperthermophilic archaea, thriving in conditions over 50 °C with enriched metal ions, and is thus dramatically different from bacteria^21^. Moreover, the components of the rRNA operon differ across archaeal species. For example, *H. volcanii* (Euryarchaea, Halobacteriales) has two 16S–23S–5S rRNA operons, whereas *P. furiosus* has a single 16S–23S rRNA operon and two independent 5S rRNA genes (Fig. 1A). Therefore, rRNA biogenesis has not yet been clarified in the Archaea.

The aim of the present study is to elucidate whether the FAU-1 possesses an RNase activity because of the partial similarity between FAU-1 and RNase E. The similarity of the regions involved in RNA recognition in FAU-1 and RNase E suggests that the mechanism that allows FAU-1 to recognize its target substrates might be similar to that of RNase E. To test this hypothesis, we purified recombinant FAU-1 protein, and assessed the RNA degradation activity using precursor 5S rRNA (pre-5S rRNA) which is known to be a substrate of RNase E. The *in vitro* results show the pre-5S rRNA is degraded by FAU-1 addition in a time and dose dependent manner. Furthermore, to investigate the roles of FAU-1 *in vivo*, we constructed *fau-1* gene deletion mutant strains of *Thermococcus kodakarensis*, which had significant growth defects with different lengths of rRNAs. We propose that FAU-1 is a novel RNase which possesses an activity to degrade and/or process precursor rRNA.

## Results

### Sequencing analysis of 5′ precursor 5S rRNA sequences in *P. furiosus*

In *E. coli* RNase E, the S1 subdomain and 5′ sensor subdomain are crucial for its recognition of RNA^3,4^. Because each of these subdomains is predicted by the Pfam database (http://pfam.xfam.org) to be present in *P. furiosus* FAU-1 (Fig. S1A), we reasoned that *P. furiosus* FAU-1 might recognize the substrates of RNase E. We focused on pre-5S rRNA, one of the major RNA substrates of RNase E. The *P. furiosus* genome contains two 5S rRNA genes (*PF_r003* and *PF_r004*) (Fig. 1A), but the pre-5S rRNA sequences have not yet been identified in a number of Archaea, including *P. furiosus*. To identify the *P. furiosus* pre-5S rRNA sequences, a cDNA library was prepared from *P. furiosus* RNA, size-fractionated to approximately 120–500 nucleotides (nt), and was sequenced using a 5S-rRNA-specific primer, as described in the *Experimental procedures*. Hereafter, we refer to *P. furiosus* or *T. kodakarensis* rRNA as “pfu XX rRNA” (e.g., pfu 5S rRNA) or “tko XX rRNA” (e.g., tko 16S rRNA), respectively, and to *P. furiosus* or *T. kodakarensis* protein as “pfu XXX” or “tko XXX”, respectively. The sequencing data indicated the presence of 63- and 54-nt sequences corresponding to the upstream regions of the *PF_r003* and *PF_r004* genes, respectively (Fig. 1B). These sequences were considered to be the 5′ precursor sequences of 5S rRNA. We then compared the nucleotide sequences of the pfu 5′ pre-5S rRNAs with those of upstream regions in the 5S rDNA in closely related species. We found an AU-rich sequence 27–39 nt upstream from the mature pfu 5S rRNA that was highly conserved in members of the Thermococcales, including *Thermococcus* and *Pyrococcus* (Fig. 1C). In *P. furiosus*, two promoter regions (approximately 30 and 60 nt upstream from the mature 5S rRNA coding site) were predicted in each 5S rRNA gene with the UCSC Archaeal Genome Browser database (http://archaea.ucsc.edu)^22,23^. Our results suggest that pre-5S rRNA is transcribed from a promoter approximately 60 nt upstream from the mature 5S rRNA coding site.

### pre-5S rRNA is degraded by the reaction with FAU-1 ***in vitro***

**Figure 2:**
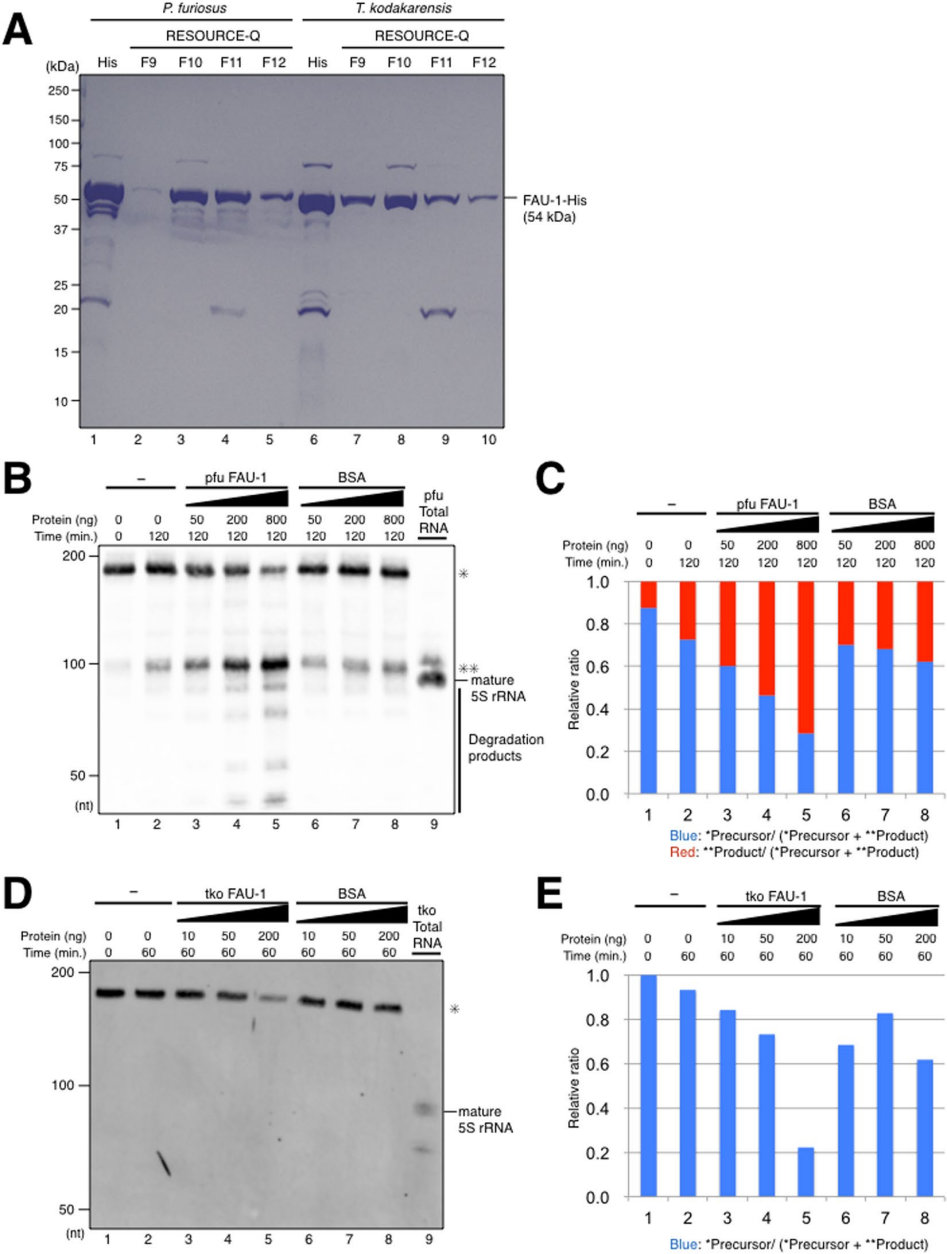
Degradation of 5′ precursor 5S rRNAs by FAU-1 proteins of *P. furiosus* and *T. kodakarensis in vitro*. (**A**) Purification of pfu and tko FAU-1s. FAU-1 proteins were initially purified with a His affinity column (His), and then with a RESOURCE-Q ion-exchange column (fractions 9–12 [F9–F12]). Each F10 was used for the experiments described below. (**B**) Degradation assay using pfu FAU-1. *In vitro* transcripts of the pfu 5′ pre-5S rRNA (0.4 μg, approximately 4 pmol), including 60 nt upstream from the mature pfu 5S rRNA (indicated by a single asterisk), were incubated with different concentrations of pfu FAU-1 for 120 min at 65 °C and then analyzed with northern blotting. Total *P. furiosus* RNA was also analyzed as a positive control for mature 5S rRNA. BSA was used as the negative control against pfu FAU-1. Double asterisks indicate the main accumulated products of FAU-1 degradation. The positions of the RNA size markers are shown on the left. (**C**) Relative ratio of the precursor (*) and accumulated products (**) in Fig. 2B. Average (N = 2) of the relative ratio was calculated from the intensity of precursors (*) and products (**). (**D**) Degradation assay using tko FAU-1. *In vitro* transcripts of the tko 5′ pre-5S rRNA (0.4 μg, approximately 4 pmol), including 60 nt upstream from the mature tko 5S rRNA (indicated by a single asterisk), were incubated with different concentrations of tko FAU-1 for 60 min at 52 °C. (**E**) Relative ratio of the precursor (*) in Fig. 2D. Relative ratio was calculated from the intensity of precursors (*).

To test whether *P. furiosus* FAU-1 has RNase activity for pre-5S rRNA, we performed an *in vitro* degradation assay using purified pfu FAU-1 protein from fraction 10, shown in Fig. 2A (lane 3), and the pfu 5′ precursor 5S rRNA (186 nt). The RNA includes the mature 5S rRNA sequence (*PF_r003*, 126 nt) and the 60 nt upstream sequence. Hereafter, we refer to this 5′ precursor 5S rRNA substrate as the “pfu 5′ pre-5S rRNA”. To eliminate RNase and metal ion contaminations derived from *E. coli*, the purified FAU-1 was heated for 15 min at 85 °C, and was dialyzed twice with the buffer including 1 mM EDTA and 7 mM 2-mercaptoethanol in the process of purification (see *Experimental Procedures*). The transcribed RNA was purified on a denaturing polyacrylamide gel containing urea, and pfu 5′ pre-5S rRNA was excised and extracted from the gel. To mimic physiological conditions, the pfu 5′ pre-5S rRNA substrate was incubated at 65 °C in the presence or absence of pfu FAU-1, after which the samples were subjected to a northern blot analysis. The data show that the degradation of pfu 5′ pre-5S rRNA occurred in the presence of pfu FAU-1, and the degree of degradation correlated with the amount of pfu FAU-1 present (Fig. 2B and C, lanes 3–5, single asterisk). Degradation products of pfu 5′ pre-5S rRNA accumulated with increasing amounts of pfu FAU-1 (Fig. 2B and C, lanes 3–5, double asterisks). Despite this, no band corresponding to the exact size of mature pfu 5S rRNA was detected in samples treated with pfu FAU-1 (Fig. 2B, lanes 3–5, double asterisk, and lane 9), suggesting that other proteins are required to reproduce the complete maturation of 5S rRNA seen *in vivo*. The short degradation products detected with increasing amounts of pfu FAU-1 suggest that pfu FAU-1 has endoribonuclease activity, degrading 5′ pre-5S rRNA at various locations within its sequence (Fig. 2B, vertical line). Note that a slight increase in the 100-nt 5S rRNA degradation product was observed with heating over time and/or in the bovine serum albumin (BSA) control samples (Fig. 2B and C, lanes 1–2, and 6–8). However, the degree of degradation was not dependent on the level of BSA protein, suggesting that some nonspecific degradation occurred under these assay conditions. We also applied a higher temperature condition of 80 °C, and the results showed that pre-5S rRNA was degraded by FAU-1. On the other hand, experiments carried out under a lower temperature condition of 37 °C did not show any degradation activity (Fig. S2B and C). Additionally, we tested whether FAU-1 has degradation preferences of pre-5S rRNA using substrates containing pre-5S rRNA and yeast mature tRNA (Fig. S2D and E). The data suggested that the FAU-1 preferentially degrade pre-5S rRNA more than yeast tRNA control. The apparent degradation velocity of pfu FAU-1 against pre-5S rRNA is ~0.09 pmol/min/μg protein in this condition.

To determine whether FAU-1 has similar activity in other closely related species, we purified the FAU-1 protein from *T. kodakarensis* (Fig. 2A, lanes 6–10) and tested its activity in the *in vitro* degradation assay, as for pfu FAU-1. The precursor 5S rRNA probe substrate (186 nt), which included the mature 5S rRNA (*Tk_r01*, 126 nt) and its 60-nt upstream sequence, was transcribed and incubated in the presence or absence of tko FAU-1. The data show that the tko pre-5S rRNA decreased in a tko-FAU-1-dependent manner at 52 °C, but no processed rRNA species were detected with this protein (Fig. 2D and E, lanes 3–5). We also performed the same experiment at different temperature conditions, and degradation by tko FAU-1 was observed within a 46–65 °C temperature range (Fig. S2F and G). The apparent degradation velocity of tko FAU-1 against pre-5S rRNA is ~0.13 pmol/min/μg protein in this condition. The results raise the possibility that FAU-1 recognizes the pre-5S rRNA as a substrate for degradation in the Thermococcales.

### FAU-1 preferentially cleaves UA sequences in pre-5S rRNA

**Figure 3:**
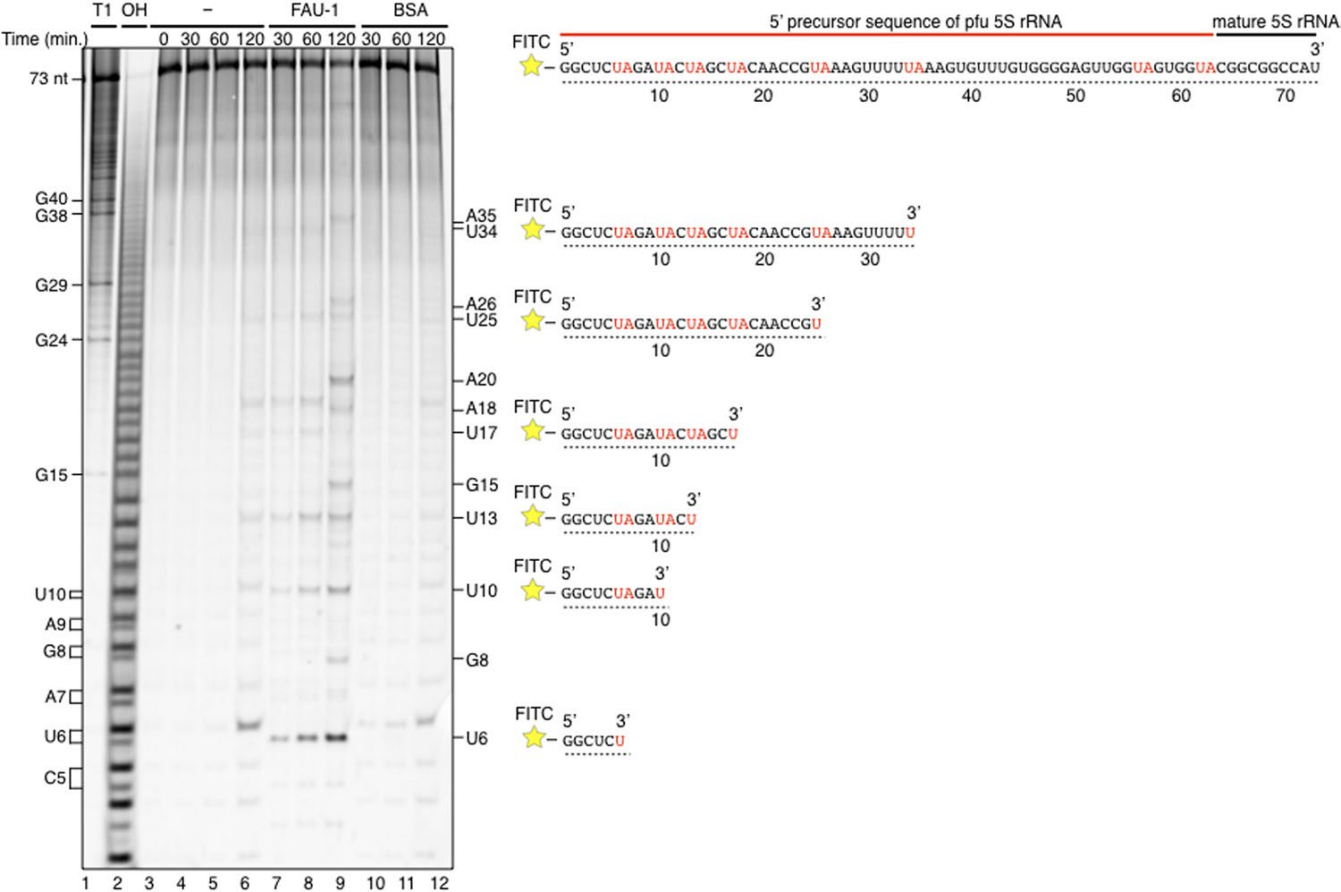
*Pyrococcus furiosus* FAU-1 preferentially cleaves UA sequences in the 5′ precursor region of 5S rRNA. Five pmol of short RNA probe (73 nt) corresponding to part of the 5′ pre-5S rRNA, labeled with FITC at the 5′ end, was incubated with 800 ng of pfu FAU-1 through a time course. The degradation products of this RNA probe were analyzed on a 20% acrylamide–8 M urea gel (*left*). BSA was used as the negative control. The RNA probe was partially digested with RNase T1 or NaOH for sequence determination (lanes 1–2). Nucleotide sequences of typical degradation products of the RNA probe produced by FAU-1 are shown on the right. UA sequences in the RNA probe are shown in red.

To investigate the site-specificity of the pfu FAU-1 activity for pre-5S rRNA, a short RNA probe (73 nt) corresponding to the pre-5S rRNA was labeled at its 5′ end with fluorescein isothiocyanate (FITC) and incubated with pfu FAU-1 at 65 °C *in vitro*. Specific FAU-1 degradation products were consistently observed, cleaved at UA sequences in the probe, generating fragments ending in U (Fig. 3; U6, U10, U13, U17, U25, and U34), suggesting that pfu FAU-1 has UA-sequence-specific degradation activity. However, the residue immediately 5′ to the mature 5S sequences is an A, suggesting that additional activity may be required in addition to FAU-1 for 5S rRNA maturation. It is noted that some of the shorter fragments in the alkaline-fragmented control lane migrated as doublets (Fig. 3, lane 2). One possibility regarding this finding is due to a terminal 2′,3′-cyclic phosphate at the 3′ end of RNAs after degradation by FAU-1. It is consistent with reports that a terminal 2′,3′-cyclic phosphate at the 3′ end of RNAs is generated by some RNases, such as RNase I. Typically, RNAs with a terminal 2′,3′-cyclic phosphate migrate faster than the linear phosphate species on a denaturing gel^24,25^. Importantly, the pfu FAU-1 degradation products cleaved at positions U6 or U10 (Fig. 3, lanes 7–9) migrated faster than those in the negative control (Fig. 3, lanes 4–6 and 10–12). To test this hypothesis, we investigated whether bacterial alkaline phosphatase (BAP) treatment affects the electromobility of degradation products by FAU-1 or not. As a result, the electromobility of degradation products by FAU-1 did not change by phosphatase treatment, suggesting that FAU-1 degrades the pre-5S RNA sequences with formation of 2′,3′-cyclic phosphate and 5′hydroxyl termini (Fig. S3A and B).

### Addition of Mg^2+^ ions promote the first-cut of pre-5S rRNA by FAU-1

**Figure 4:**
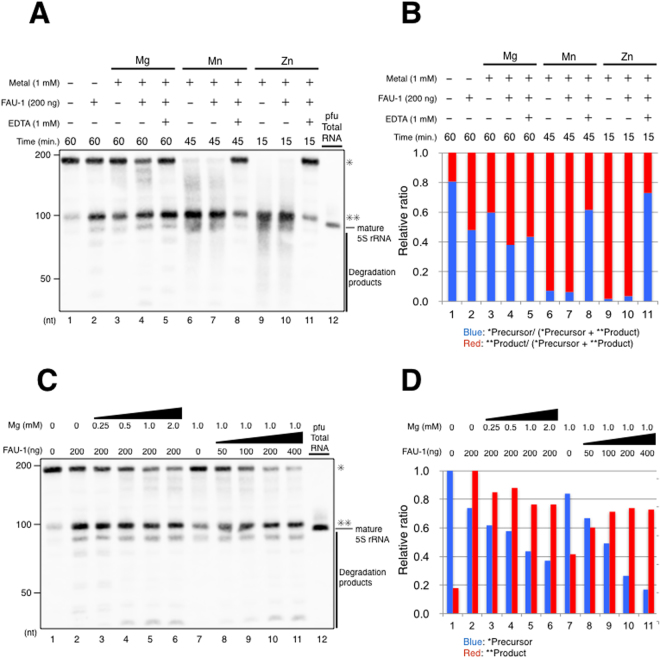
Addition of Mg^2+^ ion promote degradation activity of the FAU-1 at first-cut of pre-5S rRNA. (**A**) Effects of divalent metal ions on the degradation of 5′ pre-5S rRNA by pfu FAU-1. The pfu 5′ pre-5S rRNA substrate (0.2 μg, approximately 2 pmol indicated by a single asterisk) was incubated with each divalent metal ion (Mg^2+^, Mn^2+^, or Zn^2+^) in the presence or absence of pfu FAU-1 at 65 °C. EDTA was used to chelate the divalent metal ions (lanes 5, 8, and 11). A northern blotting analysis was then performed against those samples. Total *P. furiosus* RNA was also analyzed as the positive control for mature 5S rRNA. Double asterisks indicate the accumulated degradation products generated by FAU-1. The positions of the RNA size markers are shown on the left. (**B**) Relative ratio of the precursor (*) and accumulated products (**) in Fig. 4A. Relative ratio was calculated from the intensity of precursors (*) and products (**). (**C**) Effects of the Mg^2+^ ion concentration on the degradation of 5′ pre-5S rRNA by pfu FAU-1. The 5′ pre-5S rRNA substrate (0.2 μg, approximately 2 pmol) was incubated with different concentrations of Mg^2+^ ions and of FAU-1 for 60 min at 65 °C. (**D**) Relative ratio of the accumulated precursor (*) or products (**) in Fig. 4C. Relative ratio was calculated from the intensity of precursors (*) or products (**), independently.

A divalent metal ion is typically required for RNase activity, and the activity of RNase E is reportedly dependent on Mg^2+^ and/or Mn^2+^ ions^17,19^. A Zn^2+^ ion is also required for RNase E to form a tetrameric structure, and consequently indirectly affects its catalytic activity^18^. Therefore, we tested whether these divalent metal ions affect the pfu FAU-1 RNase activity for pre-5S rRNA *in vitro*. In this experiment, the addition of Mg^2+^ to pfu FAU-1 caused additive effects for degradation activity for pre-5S rRNA compared with treatment with pfu FAU-1 alone or Mg^2+^ alone (Fig. 4A and B, lanes 1–4). Note that strong degradation of the pre-5S rRNA was observed with the addition of Mn^2+^ or Zn^2+^ ions alone, but not with Mg^2+^ alone (Fig. 4A and B, lanes 6 and 9). The addition of Mn^2+^ to pfu FAU-1 weakly enhanced its RNase activity for pre-5S rRNA compared with the addition of only pfu FAU-1 or only Mn^2+^ (Fig. 4A and B, lanes 6–7, and Fig. S4). However, we could not evaluate whether FAU-1 and the Mn^2+^ or Zn^2+^ ions acted in a coordinated manner in the degradation of pre-5S rRNA, because the RNA probe was rapidly degraded by the Mn^2+^ or Zn^2+^ ions (Fig. 4A and B, lanes 6–7 and 9–10, and Fig. S4). It has been reported that Mn^2+^ or Zn^2+^ ions have nonspecific degradation capacities for RNA, and this could affect the stability of pre-5S rRNA^26,27^. We also performed the degradation assay under the same conditions with ethylenediaminetetraacetic acid (EDTA), a chelating agent for divalent metal ions. The degradation capacity of FAU-1 for pre-5S rRNA in the presence of EDTA was comparable to that of FAU-1 alone (Fig. 4A and B, lanes 1–5). To analyze the effects of Mg^2+^ on FAU-1 activity in more detail, we examined the degradation of pre-5S rRNA after the titration of Mg^2+^ or the FAU-1 protein. The degradation of pre-5S rRNA by FAU-1 increased in an Mg^2+^- or FAU-1-dependent manner (Fig. 4C and D). However, the main product was slightly reduced in the presence of Mg^2+^ ions compared to FAU-1 alone (Fig. 4C and D). Also, the pattern of accumulation of degradation products changed in the presence of the Mg^2+^ ion (Fig. 4C and D, vertical line). The apparent degradation velocity of pfu FAU-1 with 1 mM of Mg^2+^ ions against pre-5S rRNA is ~0.12 pmol/min/μg protein and it is higher than that with no addition of Mg^2+^ ions. These data suggest that the effect of Mg^2+^ ions for RNase activity of FAU-1 is enhancing the first-cut of 5′ pre-5S rRNA and changing the cutting pattern.

### Degradation activity for pre-5S rRNA reduced in a series of FAU-1 mutants

**Figure 5:**
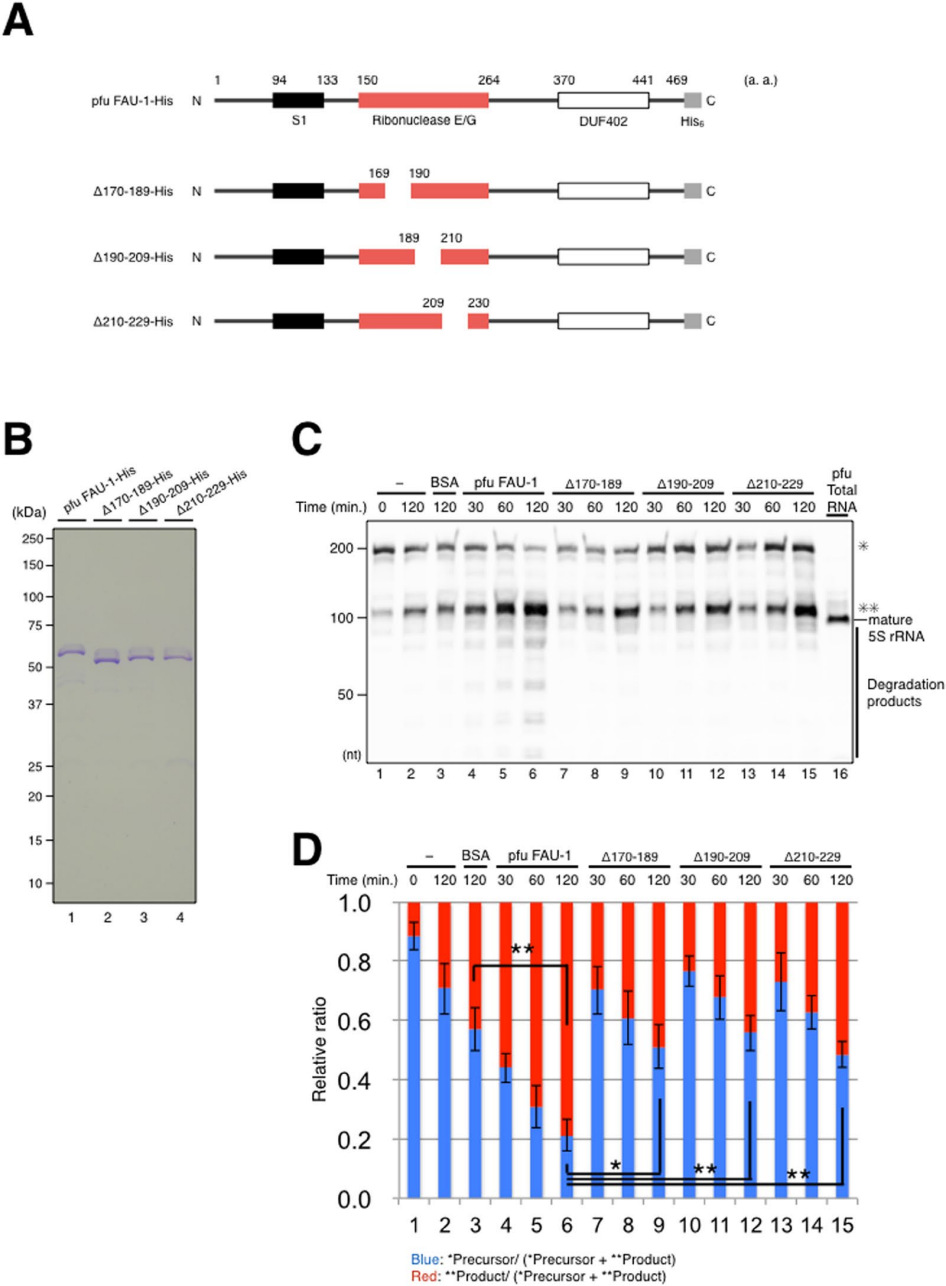
Effects of mutant *P. furiosus* FAU-1 proteins on the degradation of 5′ pre-5S rRNA. (**A**) Schematic representations of internally deleted pfu FAU-1 proteins. Bar diagrams indicate the domain information for wild-type pfu FAU-1 and the internally deleted pfu FAU-1 constructs. The S1 RNA-binding domain, ribonuclease E/G domain, unknown function DUF402 domain, and His_6_ tag are represented with black, red, white, and gray boxes, respectively. Amino acid residues 170–189, 190–209, or 210–229 in pfu FAU-1 were deleted in the different constructs. (**B**) Purification of internally deleted pfu FAU-1 proteins. WT and mutant proteins were purified (lanes 1–4) with a His affinity column and RESOURCE-Q column, as indicated in Fig. 2A. Analysis of peak fractions from RESOURCE-Q column chromatography were performed by SDS-PAGE with CBB staining. (**C**) Degradation assay using the internally deleted pfu FAU-1 proteins. The 5′ pre-5S rRNA substrates (0.2 μg, approximately 2 pmol) were incubated with WT pfu FAU-1 or the mutant proteins (800 ng each) at 65 °C over a time course, and then subjected to a northern blotting analysis. The single asterisk and double asterisks indicate the 5′ pre-5S rRNA and predominantly accumulated product, respectively. The positions of the RNA size markers are shown on the left. (**D**) Relative ratio of the precursor (*) and accumulated products (**) in Fig. 5C. The graph shows average (N = 3) and standard deviation (SD) of relative ratio calculated from the intensity of precursors (*) and products (**). The data are averaged from four independent experiments (*p < 0.05, **p < 0.01, student’s t-test).

Our Pfam analysis (http://pfam.xfam.org) predicted three domains, including the RNase E/G domain, in pfu FAU-1 (Fig. S1). The amino acid identity between the predicted RNase E/G domain in *E. coli* and that in pfu FAU-1 is 7%, whereas the similarity is approximately 23%. To test whether the RNase E/G domain of pfu FAU-1 is related to its RNase activity, plasmids expressing three histidine (3×His)-tagged FAU-1 were constructed in which pfu FAU-1 amino acid residues 170–189 (FAU-1-Δ170–189–His), 190–209 (FAU-1-Δ190–209–His), or 210–229 (FAU-1-Δ210–229–His) were deleted (Fig. 5A). These internally deleted regions are parts of the predicted pfu FAU-1 RNase E/G domain and are highly conserved in closely related species (Fig. S1). The recombinant mutant proteins were purified using the same procedure of wild-type FAU-1 (Fig. 5B), and the effects of the internal deletions were determined using the *in vitro* degradation assay. The RNA degradation activities of the internally deleted FAU-1 mutants were reduced compared with that of WT FAU-1 (Fig. 5C), and some of the smaller degradation products produced by WT FAU-1 were not produced by the internally deleted FAU-1 mutants (Fig. 5C, degradation products). However, pre-5S rRNA processing was still observed after treatment with all the variant proteins. Importantly, it should be noted that the FAU-1 mutants displayed changes in activity although the purification processes were identical, suggesting that the RNase activities observed in our experiments were due to the FAU-1 protein(s), and not contaminating proteins from *E. coli*. Here, we propose that pfu FAU-1 possesses an RNase activity, but which differs in characteristics to those from RNase E of *E. coli*. Conserved regions in the RNase E/G domain are related to its RNase activity, though a core domain involved in its RNase activity may be located in other parts.

### Deletion of the fau-1 gene in *T. kodakarensis* showed that FAU-1 plays critical roles in cell viability and rRNA maturation

**Figure 6:**
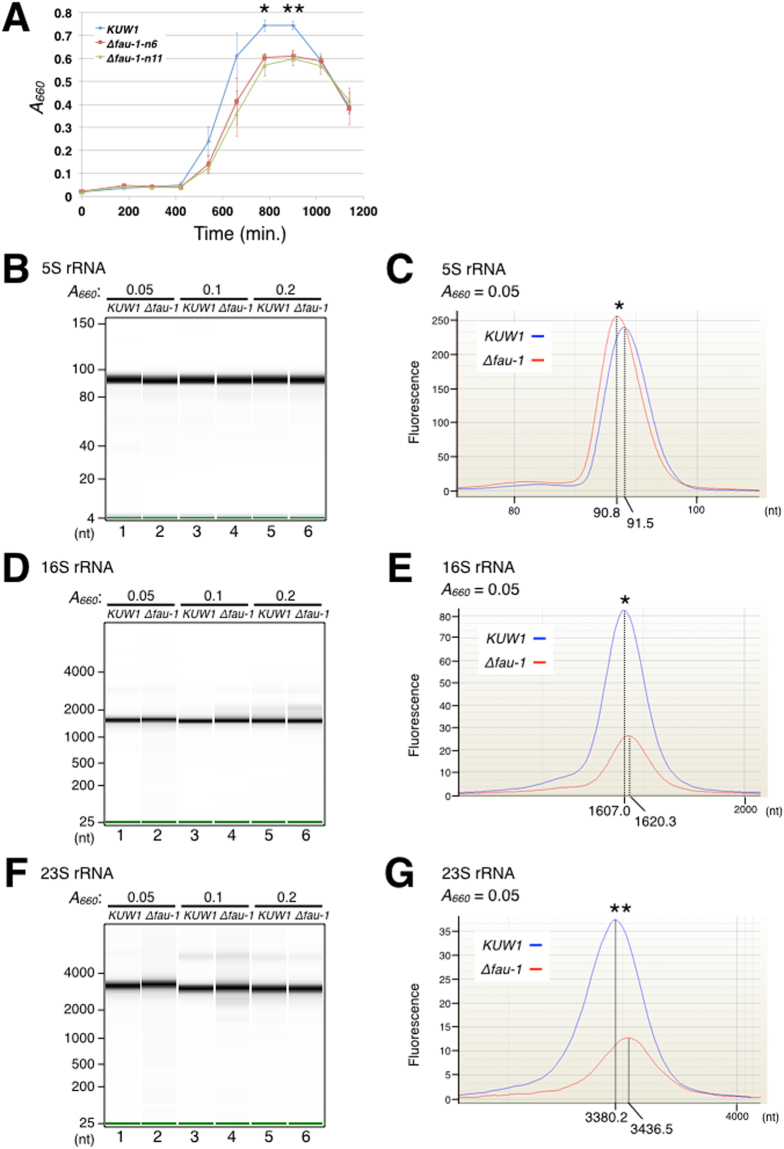
Analysis of the *fau-1* knockout strains of *T. kodakarensis*. (**A**) Growth curves for tko KUW1 cells and *fau-1* gene deletion mutants. The growth curve for tko KUW1 is shown in blue and those for the two independent *fau-1* deletion mutant clones (Δ*fau-1-n6* and Δ*fau-1-n11*) are shown in red and green, respectively. The cells were grown in ASW-YT-Mdx-W medium at 93 °C with technical triplicate (*p < 0.05, **p < 0.01, student’s t-test). (**B**), (**D**), and (**F**) Electrophoresis of rRNAs prepared from KUW1 or Δ*fau-1* cells. rRNAs were harvested at OD_660_ 0.05, 0.1, and 0.2 from both KUW1 and Δ*fau-1* cells. The lengths of 5S rRNA (**B**), 16S rRNA (**D**), and 23S rRNA **(F**) were compared between KUW1 and Δ*fau-1* cells at each OD with an Agilent 2100 Bioanalyzer (Agilent Technologies). The positions of the RNA size markers are shown on the left. (**C**), (**E**), and (**G**) Profile plot of each rRNA at OD_660_ 0.05. The y-axis represents the fluorescence intensity and the x-axis represents the nucleotide lengths. The peak of each rRNA is shown in blue (KUW1) or red (Δ*fau-1*). The data of nucleotide sizes are averaged from six biologically independent experiments (*p < 0.05, **p < 0.01, student’s t-test).

To clarify the role of FAU-1 *in vivo*, we deleted the *fau-1* gene in *T. kodakarensis*, a species closely related to *P. furiosus* and for which the genome is easy to manipulate and tools well-established^28,29^. Among the Δ*fau-1* strains, growth defects were observed at 93 °C in two independent gene disruptants (Fig. 6A, KUW1, Δ*fau-1-n*6 and Δ*fau-1-n*11). Since rRNA production or stability are major factors in proliferation ratios, and our *in vitro* data suggested the relationship between FAU-1 and rRNA processing or degradation, we hypothesized that the cause of the growth defects was due to the rRNA status in *fau-1* deletion mutant. To test this hypothesis, we extracted the RNA samples from KUW1 and Δ*fau-1* cells that were cultured and their growth was monitored as the optical density at 660 nm (OD_660_). The cells were harvested at OD_660_ 0.05, 0.1, and 0.2, and the total RNA was extracted. The rRNA, normalized to the total RNA amount, was separated by electrophoresis on a denaturing agarose gel, and was detected with SYBR Green II staining. The electromobility of the 16S and 23S rRNAs differed between the KUW1 and Δ*fau-1* cells (Fig. S5A). To eliminate the effects of contaminants in a more detailed analysis, the 5S, 16S, and 23S rRNAs were purified with denaturing gel electrophoresis and then separated with a bioanalyzer. Consistent with the SYBR Green II analysis, the electromobility of the rRNAs from the Δ*fau-1* cells were significantly altered relative to that in the KUW1 cells (Fig. 6B–G and Table S1). The difference in electromobility of 23S rRNA at OD_660_ 0.05 corresponded to a length difference of approximately 50 nt between the KUW1 and Δ*fau-1* cells (Fig. 6F–G, Table 1). In addition, we investigated the differences in degradation products of 16S and 23S rRNA between WT and Δ*fau-1* strains. As a result, the degradation patterns, sizes and accumulation levels were different between WT and Δ*fau-1* strains (Fig. S5B). These results show that FAU-1 is critical for *T. kodakarensis* cell growth *in vivo* and suggests that FAU-1 has a role in the maturation or stability of rRNAs.

**Table 1:** Strains and plasmids used in this study.

Strain/plasmid	Relevant genotype and property	Source
**Strain**		
*Pyrococcus furiosus DSM3*6*38*	Wild type	
*Thermococcus kodakarensis KOD1*	Wild type	Atomi *et al*., 2004
*KUW1*	*KOD1* Δ*pyrF* Δ*trpE*	Sato *et al*., 2005
*KARNE1*	*KUW1* Δ*fau-1-n*6	This study
*KARNE2*	*KUW1* Δ*fau-1-n11*	This study
**Plasmid**		
pMD20	Vector	Takara bio
pET23b	Vector	Novagen
pET-pfu-FAU-1-His	Derivative of pET23b carrying *pfu fau-1-His*_*6*_	Kanai *et al*., 2003
pET-tko-FAU-1-His	Derivative of pET23b carrying *tko fau-1-His*_*6*_	This study
pET-pfu-FAU-1-Δ170-189-His	Derivative of pET23b carrying *pfu fau-1-*Δ*170-189-His*_*6*_	This study
pET-pfu-FAU-1-Δ190-209His	Derivative of pET23b carrying *pfu fau-1-*Δ*190-209-His*_*6*_	This study
pET-pfu-FAU-1-Δ210-229His	Derivative of pET23b carrying *pfu fau-1-*Δ*210-229-His*_*6*_	This study
pUC118	Vector	Takara bio
pUC-T7-pfu-pre-5S rRNA	Derivative of pUC118 carrying *pfu pre-5S rRNA* (*PF_r003*)	This study
pUC-T7-tko-pre-5S rRNA	Derivative of pUC118 carrying *tko pre-5S rRNA* (*TKr01*)	This study
pUD3	Vector	Yokooji *et al*., 2009
pUD3_2227D	Derivative of pUD3 used to make Δ*fau-1*	This study

## Discussion

In a previous study, we found that *P. furiosus* FAU-1 is an RNA-binding protein^1^. Although pfu FAU-1 binds AU-rich sequences, the function of the FAU-1 protein was unclear. In this study, we found that FAU-1 possesses RNase activity against pre-5S rRNA (Fig. 2B–C) and specifically degrades UA sequences of pre-5S rRNA that are highly conserved in Thermococcales (Figs 1 and 3). RNase E also degrades pre-5S rRNA^15^ and has degradation specificity for UA sequences^30,31^. These characteristics may explain the similarities between the RNA recognition domains of pfu FAU-1 and *E. coli* RNase E (Fig. S1). Further, Mg^2+^ ion affects RNase activity of pfu FAU-1 against pre-5S rRNA, but details remain unclear (Fig. 4). Moreover, our result show that internal deleted FAU-1 reduce their RNase activity (Fig. 5), but the catalytic center of RNase activity of pfu FAU-1 is unknown (Fig. S1). To elucidate those questions, it will require X-ray crystal structural analysis of FAU-1 in the future.

The electromobility of 5S rRNA processed by FAU-1 *in vitro* differs from that of mature 5S rRNA of *P. furiosus* generated *in vivo* (Fig. 2B). Therefore, we infer that some factors are still lacking *in vitro* that are required to generate mature 5S rRNA. In Archaea, various modifications occur at several positions in the mature 5S and 23S rRNAs^32–35^. An rRNA methylation enzyme is also associated with the stability and maturation of rRNA in *E. coli* ^36,37^. The pre-5S rRNA transcribed *in vitro* in this study was not modified by those enzymes, and their absence may explain the differences in the electromobility of the pfu 5S rRNA processed by FAU-1 *in vitro* and that of the mature pfu 5S rRNA generated *in vivo*. Other factors that act cooperatively with FAU-1 may also be lacking in the *in vitro* experiments. For example, *E. coli* RNase E forms a huge protein heterocomplex, called the RNA degradosome, consisting of RhlB RNA helicase, enolase, PNPase, and Hfq, which work in concert to degrade RNA substrates^38-40^. We did not detect any products when we added tko FAU-1 for *in vitro* degradation assay (Fig. 2D). In addition to the reasons mentioned above, the amino acid identity of pfu FAU-1 and tko FAU-1 is 74.5% and this difference may produce different activities in *in vitro* assay. Indeed, the optimal reaction temperature in *in vitro* degradation assay is different between pfu and tko FAU-1 (Fig. S2).

Our results show that FAU-1 preferentially cleaves UA sequences *in vitro*, and the migration of the pfu FAU-1 degradation products cleaved at the U6A7 and U10A11 positions were different from those of no protein or BSA control (Fig. 3). When we digested the RNA probe with NaOH, we observed that doublet bands were evident on the gel at the lower molecular weights (Fig. 3, lane 2). We infer that the difference in the two bands is attributable to the propensity of the 3′ end of a phosphate group to cyclize. The lower band represents a degradation product with a 3′-terminal 2′,3′-cyclic phosphate, whereas the upper band represents a degradation product with a linear phosphate^24^. Moreover, the electromobility of degradation products by FAU-1 did not change by phosphatase treatment (Fig. S3A). This result suggests that the FAU-1 possesses the RNase activity with formation of 2′,3′-cyclic phosphate and 5′hydroxyl termini, as do RNase I and the tRNA splicing endoribonuclease EndA^41–43^.

To clarify the roles of FAU-1 *in vivo*, we constructed and analyzed *fau-1* gene deletion mutants in *T. kodakarensis*. We observed delayed cell growth and a reduced maximal OD in the Δ*fau-1* mutant strains compared with those of the KUW1 strain (Fig. 6A). The electromobility of the 5S, 16S, and 23S rRNAs also differed between the KUW1 and Δ*fau-1* cells at OD_660_ 0.05 (Fig. 6B–G and Table S1). For example, the electromobility of the Δ*fau-1* 23S rRNA displayed a length approximately 50 nt greater than that in the KUW1 cells (Fig. 6F–G and Table S1). The reason for the difference in rRNA electromobility between the KUW1 and Δ*fau-1* cells has not been determined, but could potentially be explained by the effects of either rRNA processing and/or modification. However, our data suggest that the deletion of the *fau-1* gene affects rRNA maturation, resulting in a growth defect from the lag phase to the stationary phase. Interestingly, the differences of rRNA length at OD_660_ 0.05 disappeared at later points, and then cell proliferation started with a delay, suggesting that deletion of the *fau-1* gene might be compensated by some different mechanisms, allowing cell proliferation *in vivo*.

The successful construction of the *fau-1* gene deletion mutants indicates that the *fau-1* gene is not essential for *T. kodakarensis* cell viability. This also suggests that alternative factors are involved in 5S rRNA maturation, beside FAU-1. It has been reported in *H. volcanii* (Euryarchaea, Halobacteriales) that the maturation of the 5′ end of the 5S rRNA can be performed by tRNase Z^44^, whose predominant role is in tRNA maturation. tRNase Z is thought to recognize the tRNA-like structure in the region upstream from the 5S rRNA. However, although tRNase Z is conserved in the Thermococcales, the tRNA-like structure is not predicted in the upstream region of either pfu or tko 5S rRNAs (data not shown). EndA is also involved in the removal of rRNA introns^45–49^, and Nob1 is related to the processing of precursor 16S rRNA in the Archaea^20^. In summary, this study demonstrates that FAU-1 is important for the growth of *T. kodakarensis*, and suggests that FAU-1 acts as an RNase and is involved in the regulation of the rRNAs biogenesis and/or turnover in a coordinated manner with several RNases. Though further studies are required for elucidation of rRNA regulation by FAU-1, our findings would lead to a greater understanding of rRNA regulation in Archaea.

## Methods

### Nucleotide sequencing of *P. furiosus* precursor 5S rRNAs

*Pyrococcus furiosus* cells were harvested and suspended in equivalent amounts of RNA buffer (20 mM sodium acetate [pH 5.5], 0.5% SDS, 1 mM EDTA) and acid phenol (pH 5.2). The mixture was incubated at 60 °C for 5 min with gentle shaking. After centrifugation (14,000 rpm for 3 min at room temperature), the supernatant was re-extracted with acid phenol. Total RNA was precipitated with the addition of 2.5 volumes of ethanol to the aqueous phase and incubated for 30 min at −80 °C. The RNA precipitate was collected by centrifugation and dissolved in water. The total RNA was separated on denaturing 6% polyacrylamide gel containing 8 M urea to obtain size-fractionated RNAs (120–500 nt). A cDNA library was prepared using the small RNA Cloning Kit (Takara Bio, Otsu, Shiga, Japan), according to the manufacturer’s protocol. The 5′ precursor DNA sequences of the 5S rRNA were amplified with PCR using a 5′ adaptor primer (S0009) and 5S-rRNA-specific primers (S0010 or S0011) corresponding from nt −5 to +15 of the mature pfu 5S rRNA. The amplified DNA fragments were cloned into the TA cloning vector pMD20 (Takara Bio), and sequenced with an ABI 3130xl Genetic Analyzer (Applied Biosystems, Foster City, CA, USA) with a universal primer (M13 primer M4 5′-GTTTTCCCAGTCACGAC-3′ or M13 primer RV 5′-CAGGAAACAGCTATGAC-3′). The plasmids and primers used in this study are listed in Tables 1 and S2, respectively.

### Construction of plasmids encoding recombinant FAU-1 proteins

The construction of plasmid pET-pfu-FAU-1–His has been previously described^1^. The other plasmids (pET-tko-FAU-1–His, pET-pfu-FAU-1-Δ170–189–His, pET-pfu-FAU-1-Δ190–209–His, and pET-pfu-FAU-1-Δ210–229–His) used in this study were constructed as follows. To construct pET-tko-FAU-1–His, a DNA fragment encoding the tko *fau-1* gene (KEGG ID: TK2227) was amplified from the *T. kodakarensis* chromosomal DNA with PCR and the primer set T0005/T0006 containing *Nde*I and *Xho*I sites. The amplified fragment was digested with *Nde*I and *Xho*I, and cloned into pET-23b (Novagen, Madison, WI, USA). To construct the internally deleted pfu FAU-1–His plasmids, DNA fragments encoding the internally deleted pfu FAU-1 proteins were amplified from pET-pfu-FAU-1–His with inverse PCR^50^ with the primer sets P0032/P0031 (pET-pfu-FAU-1-Δ170–189–His), P0034/P0033 (pET-pfu-FAU-1-Δ190–209–His), and P0038/P0039 (pET-pfu-FAU-1-Δ210–229–His). The amplified fragments were self-ligated with the Mighty Cloning Reagent Set (Blunt End) (Takara Bio).

### Purification of the FAU-1 proteins

*Escherichia coli* strain BL21(DE3) was transformed with each plasmid encoding WT FAU-1 or the FAU-1 mutants. The transformants were grown in 200 ml of LB medium containing 50 μg/ml ampicillin at 37 °C to OD_600_ 0.6. After that, isopropyl β-d-thiogalactopyranoside (0.4 mM) was added to the medium, then the samples incubated overnight at 37 °C. After incubation, the cells were harvested by centrifugation (5,000 × *g* for 5 min at 4 °C) and buffer (50 mM sodium phosphate [pH 7.4], 300 mM NaCl; the recombinant proteins were extracted by sonication (3 min) with 10 mg/ml lysozyme in binding mM imidazole). These extracts were heat treated at 85 °C for 15 min to destroy most of the endogenous proteins, especially RNases, from *E. coli*, and then centrifuged at 12,000 × *g* for 10 min at 4 °C to separate the cell debris from the extracts. The supernatants were purified with an Ni^2+^ affinity chromatography column using the Proteus IMAC Mini Kit (Pro-Chem Inc., Littleton, MA, USA). The bound FAU-1 protein was washed five times with 650 μl of wash buffer (50 mM sodium phosphate [pH 7.4], 300 mM NaCl, 30 mM imidazole) and eluted from the column with 650 μl of elution buffer (50 mM sodium phosphate [pH 7.4], 300 mM NaCl, 300 mM imidazole). To obtain high-purity FAU-1 proteins, these elution fractions were further purified with RESOURCE-Q anion-exchange column chromatography (GE Healthcare, Piscataway, NJ, USA) with a linear gradient of 0–1 M NaCl in Tris-HCl buffer (50 mM Tris-HCl [pH 8.0], 1 mM EDTA, 0.02% Tween 20, 7 mM 2-mercaptoethanol, 10% glycerol). The collected fractions were dialyzed twice with Tris-HCl buffer (50 mM Tris-HCl [pH 8.0], 1 mM EDTA, 0.02% Tween 20, 7 mM 2-mercaptoethanol, 10% glycerol), and analyzed with SDS-polyacrylamide gel electrophoresis and Coomassie Brilliant Blue staining.

### *In vitro* transcription of precursor 5S rRNAs

To construct the plasmids for the *in vitro* transcription of the precursor 5S rRNAs (pUC-T7-pfu-pre-5S rRNA and pUC-T7-tko-pre-5S rRNA), a DNA fragment containing the 5S rRNA gene (*PF_r003* or *TK_r01*), including its 5′ upstream region (60 bp) with the T7 promoter sequence, was amplified from *P. furiosus* or *T. kodakarensis* genomic DNA using the primer set P0044/P0007 or T0009/T0031, respectively. The amplified fragments were cloned into pUC118 digested with *Hin*cII using the Mighty Cloning Reagent Set (Blunt End) (Takara Bio).

The pfu or tko 5′ pre-5S rRNA was transcribed with the MEGAshortscript™ T7 Transcription Kit (Thermo Fisher Scientific, Waltham, MA, USA) and the DNA fragment that was amplified from each plasmid (pUC-T7-pfu-pre-5S rRNA or pUC-T7-tko-pre-5S rRNA) with the primer set P0044/P0007 or T0009/T0031, respectively.

### *In vitro* degradation assay

The 0.4 μg or 0.2 μg of transcribed pfu 5′ pre-5S rRNA was incubated with pfu FAU-1 at 65 °C in the presence or absence of each divalent metal ion (Mg^2+^, Mn^2+^, or Zn^2+^) in 20 μl of degradation buffer (15 mM Tris-HCl [pH 8.0], 0.3 mM EDTA, 0.006% Tween 20, 2.1 mM 2-mercaptoethanol, 3% glycerol). The 0.4 μg of tko 5′ pre-5S rRNA was incubated with tko FAU-1 at 52 °C. To stop the reaction, 1 mM EDTA and 40 μl of RNA phenol were immediately added to the reaction mixture. After centrifugation, the supernatants were analyzed by 8M Urea 6% poly acrylamide gel. The 5.0 pmol of A 5′-FITC-labeled RNA probe corresponding to the 5′ precursor region of the pfu 5S rRNA sequences (shown in Fig. 3; (5′-GGCUCUAGAUACUAGCUACAACCGUAAAGUUUUUAAAGUGUUUGUGGGGAGUUGGUAGUGGUACGGCGGCCAU-3′, 73 nt; Hokkaidou System Science, Sapporo, Hokkaido, Japan) was also incubated with pfu FAU-1 at 65 °C *in vitro*. Then, the samples were analyzed by 8M Urea 20% poly acrylamide gel. The yeast mature tRNA (Invitrogen, Cat. No. 15401–029) was used for a control of *in vitro* degradation. For phosphatase reaction, the RNA samples were incubated with bacterial alkaline phosphatase (Takara bio) for 30 min at 37 °C.

### Construction of a gene disruption plasmid for TK2227

A gene disruption plasmid encoding the tko *fau-1* gene (TK2227) (designated pUD3_2227D) was constructed as follows. The TK2227 gene, together with its 5′- and 3′-flanking regions (~1 kbp), was amplified from the *T. kodakarensis* genomic DNA using the primer set FAU_A5/FAU_A3. The amplified fragment was inserted into the *Hin*cII site of pUD3, which carries the tko *pyrF* gene^51^. To remove the TK2227 ORF from the resulting plasmid, inverse PCR was performed with the primer set FAU_B5/FAU_B3. The PCR product was self-ligated to construct pUD3_2227D. The absence of unintended mutations was confirmed with DNA sequencing.

### Transformation of *T. kodakarensis*

*Thermococcus kodakarensis* strains were cultured under strictly anaerobic conditions at 85 °C, in a nutrient-rich medium (ASW-YT) composed of 0.8 × artificial seawater (0.8 × ASW), 5.0 g/l yeast extract, and 5.0 g/l tryptone. In a typical culture, the ASW-YT medium was supplemented with 2.0 g/l elemental sulfur (S^0^) immediately before inoculation (ASW-YT-S^0^ medium). A synthetic ASW-AA-S^0^ medium was also used, consisting of 0.8 × ASW, a mixture of 20 amino acids, modified Wolfe’s trace minerals, a vitamin mixture, and 2.0 g/l elemental sulfur^52^. In all cases, resazurin was added at a concentration of 0.8 mg/l, and before inoculation, Na_2_S.9H_2_O was added to the medium until it became colorless.

*Thermococcus kodakarensis* strain KUW1 (Δ*pyrF·*Δ*trpE*)^28^ was used as the host strain for gene disruption. The transformation procedures and selection methods based on uracil prototrophy and resistance to 5-fluoroorotic acid are described elsewhere^51^. To select and isolate the gene disruptant, ASW-AA-S^0^ medium was used to enrich the transformants that displayed uracil prototrophy because the transformation plasmid, pUD3_2227D, was integrated into their chromosomes. The solid media used to isolate the gene disruptants were based on ASW-YT medium supplemented with 1.0 g/l gelrite, 0.4 g/l polysulfide, and 7.5 g/l 5-fluoroorotic acid. The genotypes of the isolated transformants were analyzed with PCR and primer set FAU_C5/FAU_C3, which anneals outside of the homologous regions. Transformants whose amplified DNA products were the expected sizes were chosen, and the relevant sequences were confirmed to be as expected. The resulting *fau-1*-gene-deficient mutants were designated KARNE1 (Δ*fau-1-n*6) and KARNE2 (Δ*fau-1-n11*).

### Growth analysis

*Pyrococcus furiosus* DSM 3638 was grown anaerobically at 96 °C in *Pyrococcus/Staphylothermus* medium (as in the Handbook of Media for Environmental Microbiology, Second Edition, p. 471). *Thermococcus kodakarensis* strains KUW1, Δ*fau-1-n*6, and Δ*fau-1-n11* were also cultured under anaerobic conditions at 93 °C. ASW-YT-Mdx-W medium was used to measure growth, which is based on ASW-YT medium, supplemented with 5.0 g/l maltodextrin and 10 µM Na_2_WO_4_. Cell growth was monitored by determining the optical density at 660 nm (OD_660_).

### Northern blotting analysis

Total RNA was extracted from *P. furiosus* or *T. kodakarensis* as described above. The total RNA sample was then separated on denaturing 6% polyacrylamide gel containing 8 M urea or denaturing 1.2% agarose gel containing 3.6% formaldehyde, and blotted onto Hybond-N+ membrane (GE Healthcare) with electroblotting or capillary action. After crosslinking, the membrane was prehybridized in DIG Easy Hyb™ buffer (Roche Diagnostics, Mannheim, Germany) for 30 min at 37 °C. A biotin-labeled antisense oligodeoxynucleotide was prepared with the Biotin 3′ End DNA Labeling Kit (Pierce Biotechnology, Rockford, IL, USA), and the membrane was hybridized overnight at 37 °C in DIG Easy Hyb™ buffer containing each labelled antisense probe. The membrane was then washed at 37 °C in buffer containing 30 mM sodium citrate, 3 mM NaCl, and 0.1% SDS. The nonisotopic blots were visualized with the BrightStar® BioDetect™ Kit (Thermo Fisher Scientific) with the ECF Substrate (GE Healthcare). The images were captured with a Molecular Imager FX Pro (Bio-Rad Laboratories, Hercules, CA, USA). The band intensities were analyzed with Molecular Imager FX™ Pro. The pfu 5S rRNA or tko 5S rRNA was detected with DNA probes N0002 (5′-CTTAACTTCCGGGTTCGAAATGAGACCGGGTGTGG-3′) or N0008 (5′-CTTAACTTCCGGGGTCGAAACGAGACCGGGTGTGG-3′), respectively. Either the tko 16S rRNA or 23S rRNA was detected with specific DNA probes (200 bp, 5′terminal sequences) labeled with digoxigenin (DIG) using a PCR DIG Probe Synthesis Kit (Roche Diagnostics, Mannheim, Germany) with the PCR primer pairs: N0009 and N0010, or N0011 and N0012.

## Electronic supplementary material

Supplementary Information
