# Supplementary material for: An archaeal RNA binding protein, FAU-1, is a novel ribonuclease related to rRNA stability in Pyrococcus and Thermococcus
Source: Sci Rep. 2017 Oct 4;7:12674. doi: 10.1038/s41598-017-13062-3 (PMC5627344; doi:10.1038/s41598-017-13062-3)
Supplement: Supplementary file 1 — Supplementary Information [file 41598_2017_13062_MOESM1_ESM.doc]

An archaeal RNA binding protein, FAU-1, is a novel ribonuclease related to rRNA stability in *Pyrococcus* and *Thermococcus*

Yoshiki Ikeda^1^, Yasuhiro Okada^2^, Asako Sato^1^, Tamotsu Kanai^2^,

Masaru Tomita^1^, Haruyuki Atomi^2^ and Akio Kanai^1^

1, Institute for Advanced Biosciences, Keio University, Tsuruoka 997-0017, Japan

2, Graduate School of Engineering, Kyoto University, Kyoto 615-8510, Japan


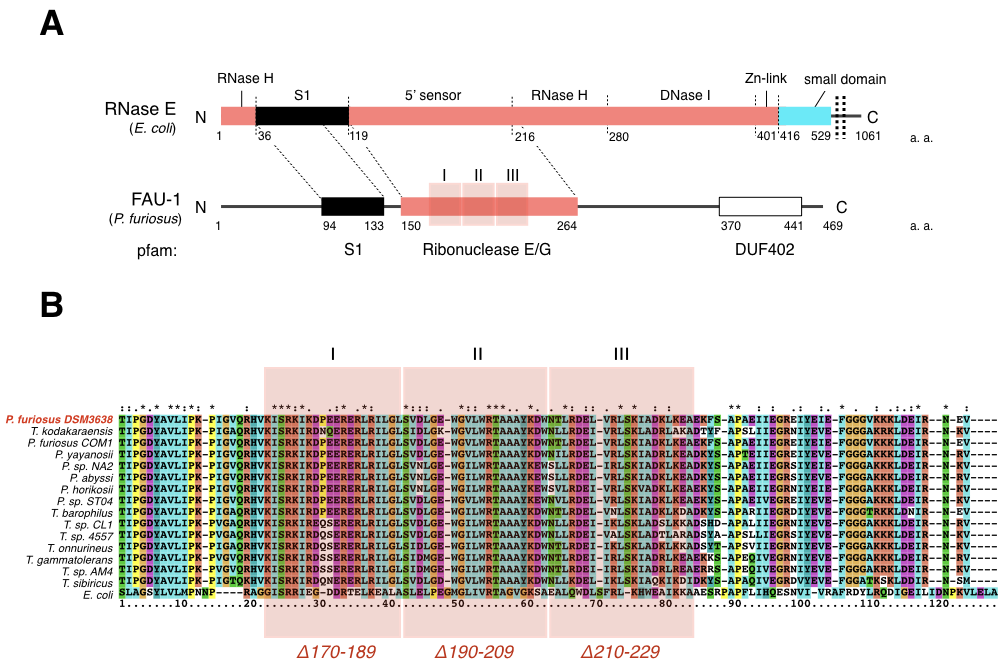


Fig. S1. Conservation of amino acid sequences in Thermococcales FAU-1 and *E. coli* RNase E. Schematic representation of the catalytic domain of the N-terminal half of RNase E in *E. coli* and the predicted domains of FAU-1 in *P. furiosus* (upper). The catalytic domain of *E. coli* RNase E is mainly divided into six subdomains and a small domain ^16^. The C-terminal half of *E. coli* RNase E is omitted by the double vertical dashed lines. Information on the predicted domains of pfu FAU-1 was obtained from the protein families database (Pfam, http://pfam.xfam.org) ^20^. The amino acid sequences of the partial ribonuclease E/G domain of FAU-1 in Thermococcales and *E. coli* RNase E (lower). Squares (I–III) indicate internally deleted regions in pfu FAU-1, as shown in Fig. 5A.


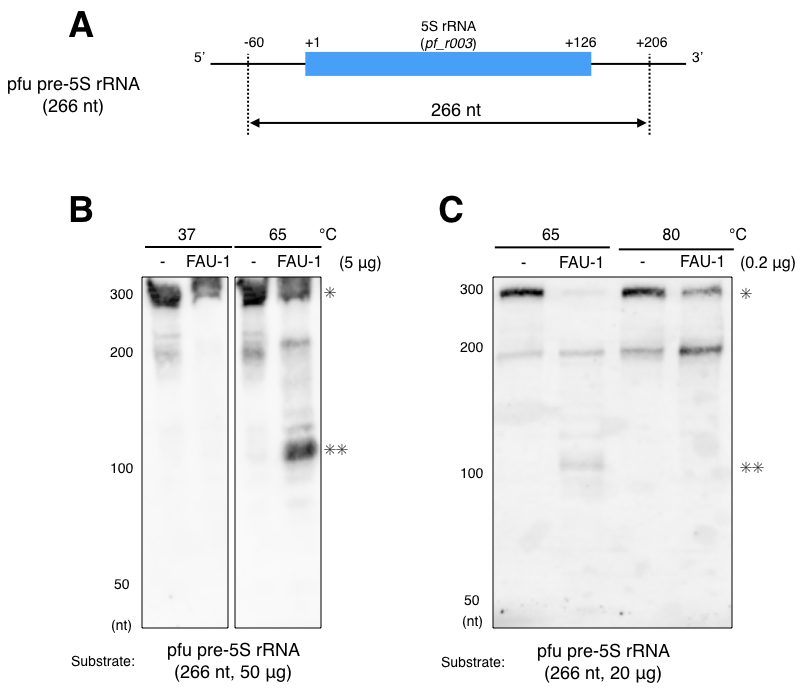

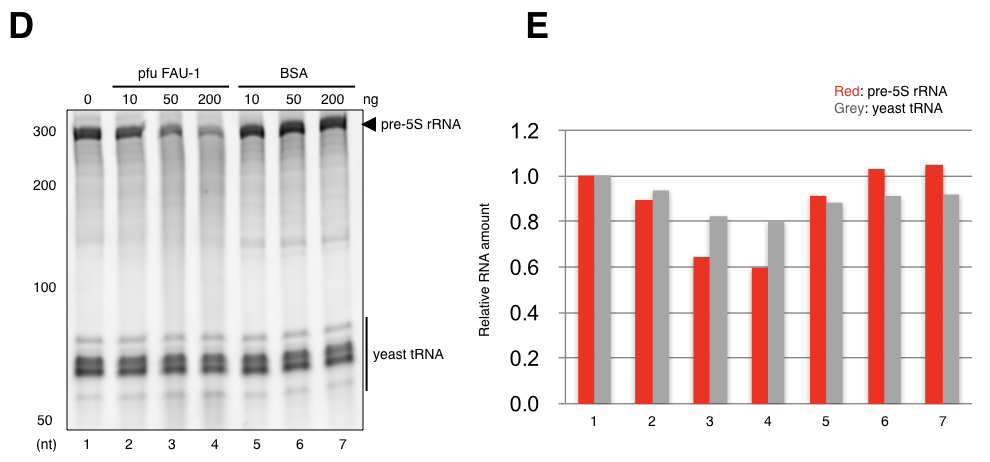

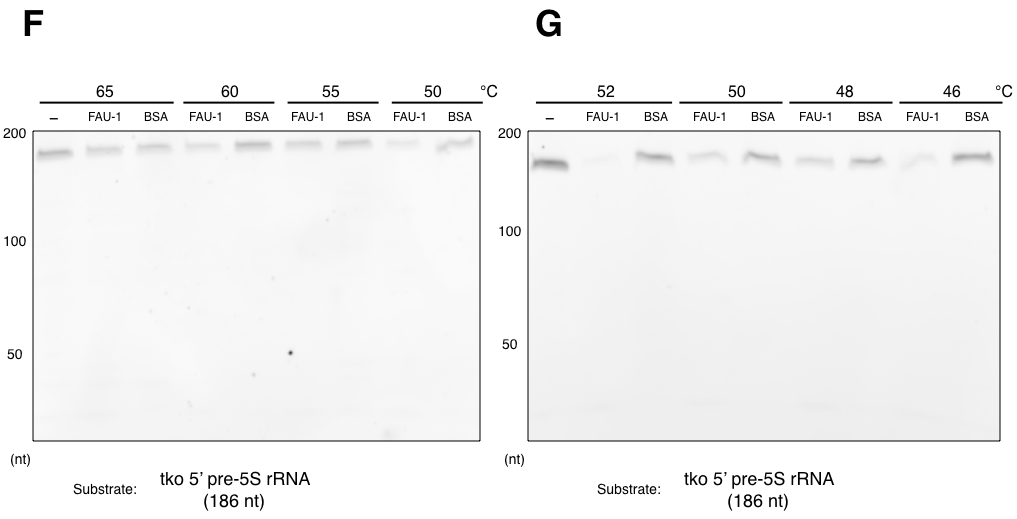


Fig. S2. (A) A schematic representation of *in vitro* transcribed RNA for degradation assay of Fig. S2B, C and D. The precursor 5S rRNA includes the mature 5S rRNA sequence (*PF_r003*, 126 nt), the 60 nt upstream sequence and the 80 nt downstream sequence. (B) and (C) Effects of temperature on the degradation activity of pfu FAU-1 against pfu precursor 5S rRNA. pre-5S rRNA substrate (single asterisk) was incubated for 1 h with pfu FAU-1 at different temperature, and the samples were then analyzed with northern blotting using pfu 5S rRNA specific DNA probe. The positions of the RNA size markers are shown on the left. Double asterisks indicate the predominant accumulation products generated by pfu FAU-1. (D) FAU-1 has degradation preference against pre-5S rRNA. To test whether the FAU-1 has degradation preference, yeast mature tRNA was mixed in the reaction mixture as a control. The samples were analyzed by SYBER Green II staining. (E) Related to (D). The graph shows relative ratio of each intensity of (E). Lane 1 is set as 1.0. (F) and (G) Effects of temperature on the degradation activity of tko FAU-1 against tko 5’ precursor 5S rRNA. 20 μg of 5’ pre-5S rRNA substrate (186 nt, single asterisk) was incubated for 1 h with 400 ng of tko FAU-1 at different temperature, and the samples were then analyzed with northern blotting using tko 5S rRNA specific DNA probe. The positions of the RNA size markers are shown on the left. Double asterisks indicate the predominant accumulation products generated by tko FAU-1.


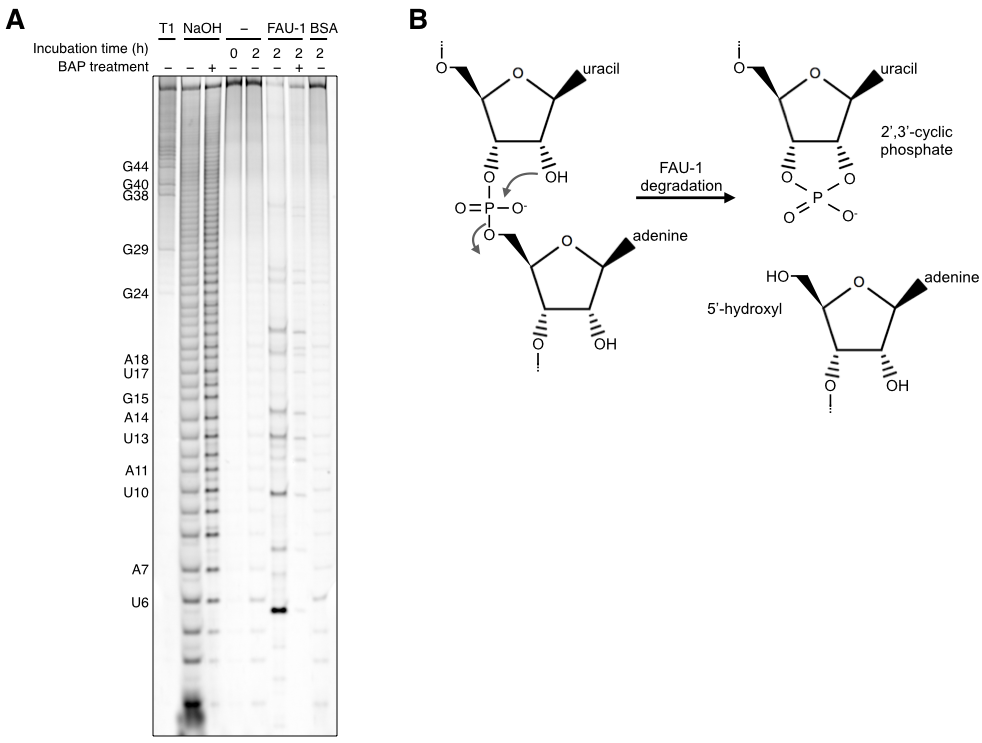


Fig. S3. (A) Phosphatase treatment do not affect the electromobility of degradation products by FAU-1. Bacterial Alkaline Phosphatase (BAP) treatment was performed after incubation with FAU-1 for 2h. Then, the degradation products of this RNA probe were analyzed on a 20% acrylamide–8 M urea gel. (B) Model of RNA degradation by FAU-1 with formation of 2’,3’-cyclic phosphate and 5’hydroxyl termini.


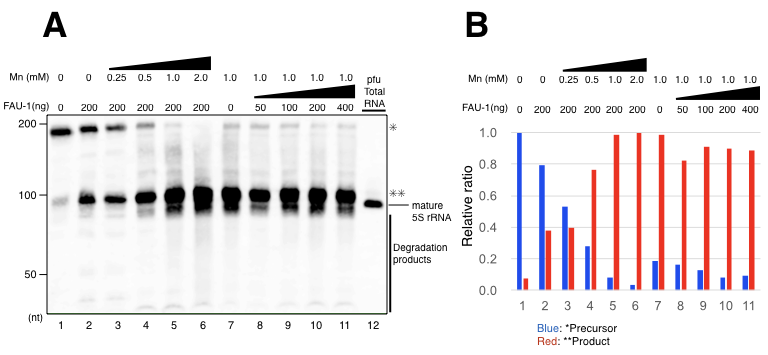


Fig. S4. Effects of Mn^2+^ ions on the degradation of precursor 5S rRNA by *P. furiosus* FAU-1. The 5′ pre-5S rRNA substrate (single asterisk) was incubated with different concentrations Mn^2+^ ions and of FAU-1, and the samples were then analyzed with northern blotting. Total *P. furiosus* RNA was also analyzed as the positive control for mature 5S rRNA. Double asterisks indicate the predominant accumulation products generated by pfu FAU-1. The positions of the RNA size markers are shown on the left.


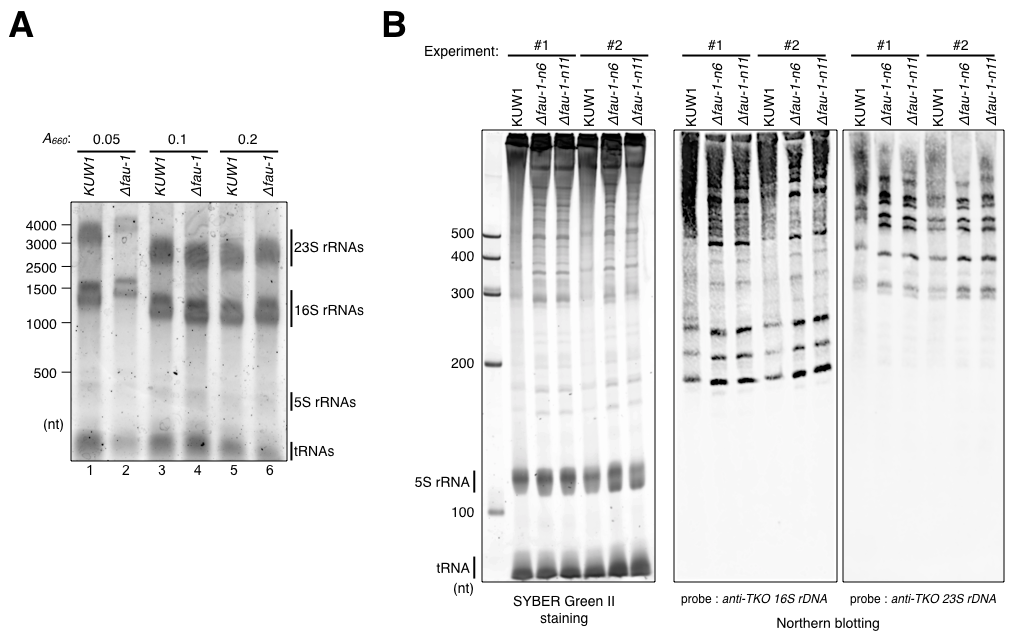


Fig. S5. (A) Differences in rRNA sizes generated in the *T. kodakarensis* strains KUW1 and *Δfau-1*. Total RNAs were extracted from KUW1 and *Δfau-1* cells at different optical densities (OD_660_ 0.05, 0.1, and 0.2), and the RNAs were separated by electrophoresis on a denaturing 1.2% agarose gel. The positions of the RNA size markers are shown on the left. (B) Effects of deletion of *fau-1* gene for rRNA degradation. 8 μg of total RNA extracted from *T. kodakarensis* WT and *Δfau-1* strains at OD660 = 0.2 were analyzed with northern blotting using either 16S or 23S specific DNA probes (200 bp, 5’ terminal sequences) labeled with digoxigenin (DIG). The experiments were performed in biological duplicates.


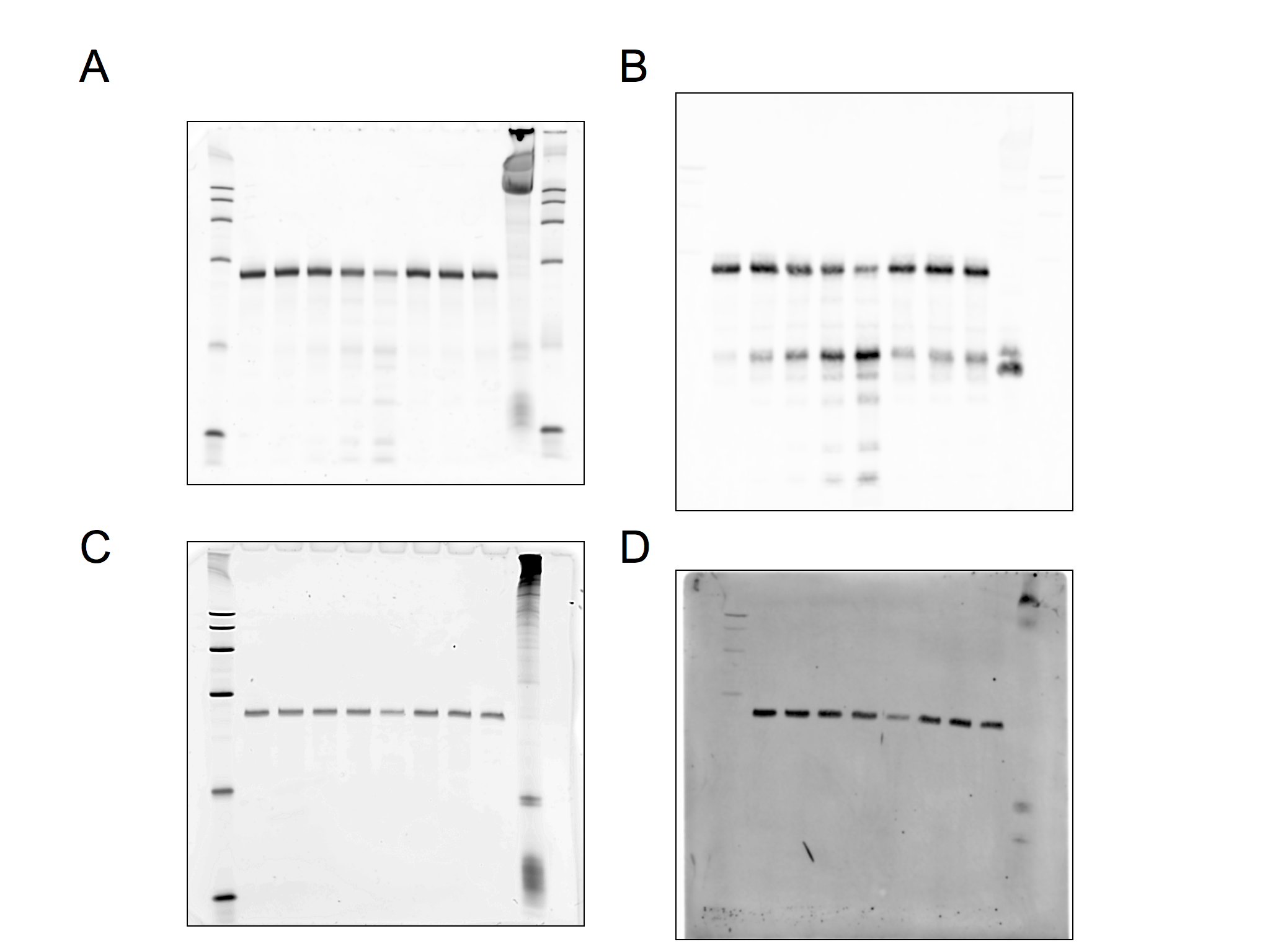


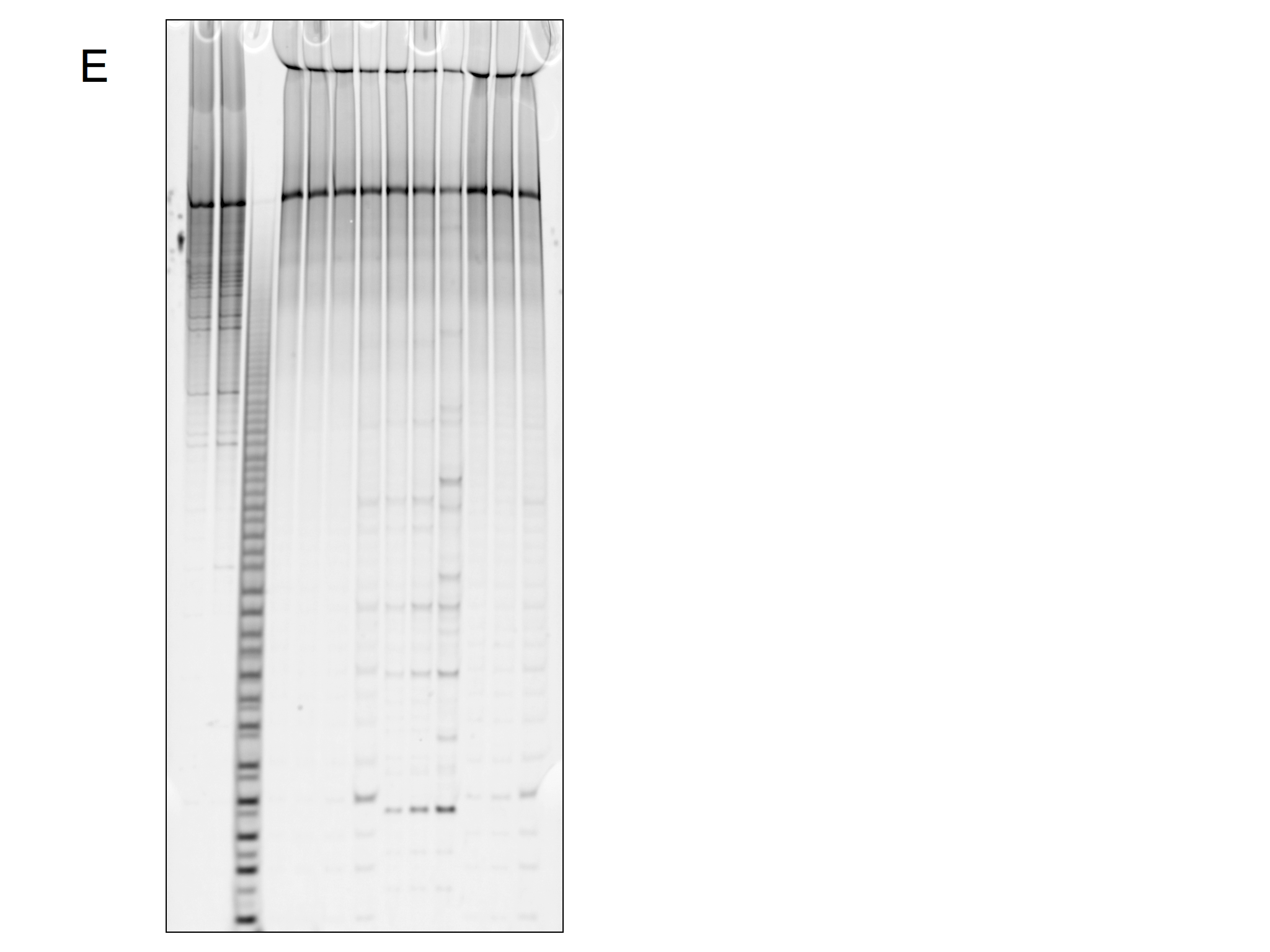


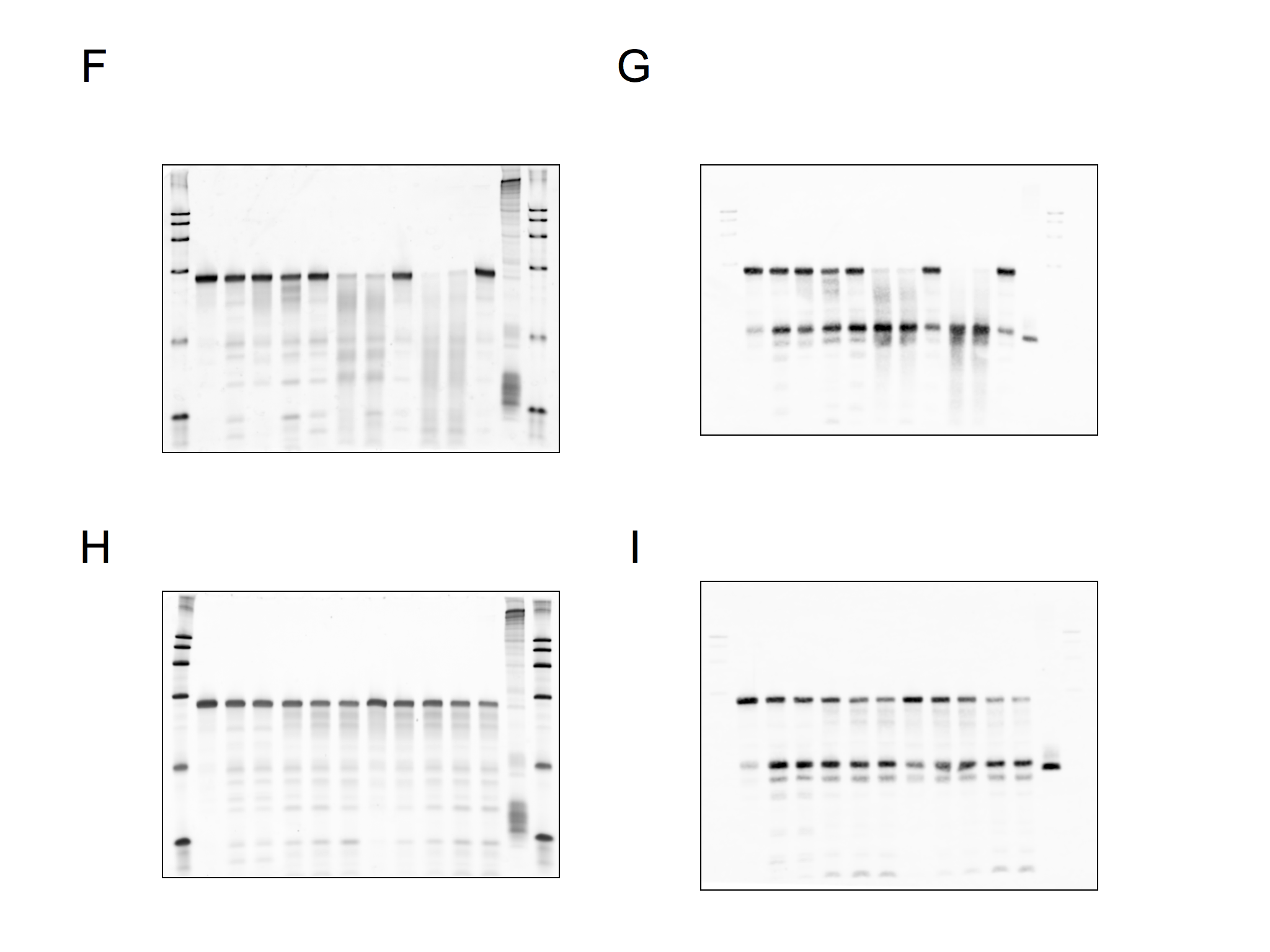


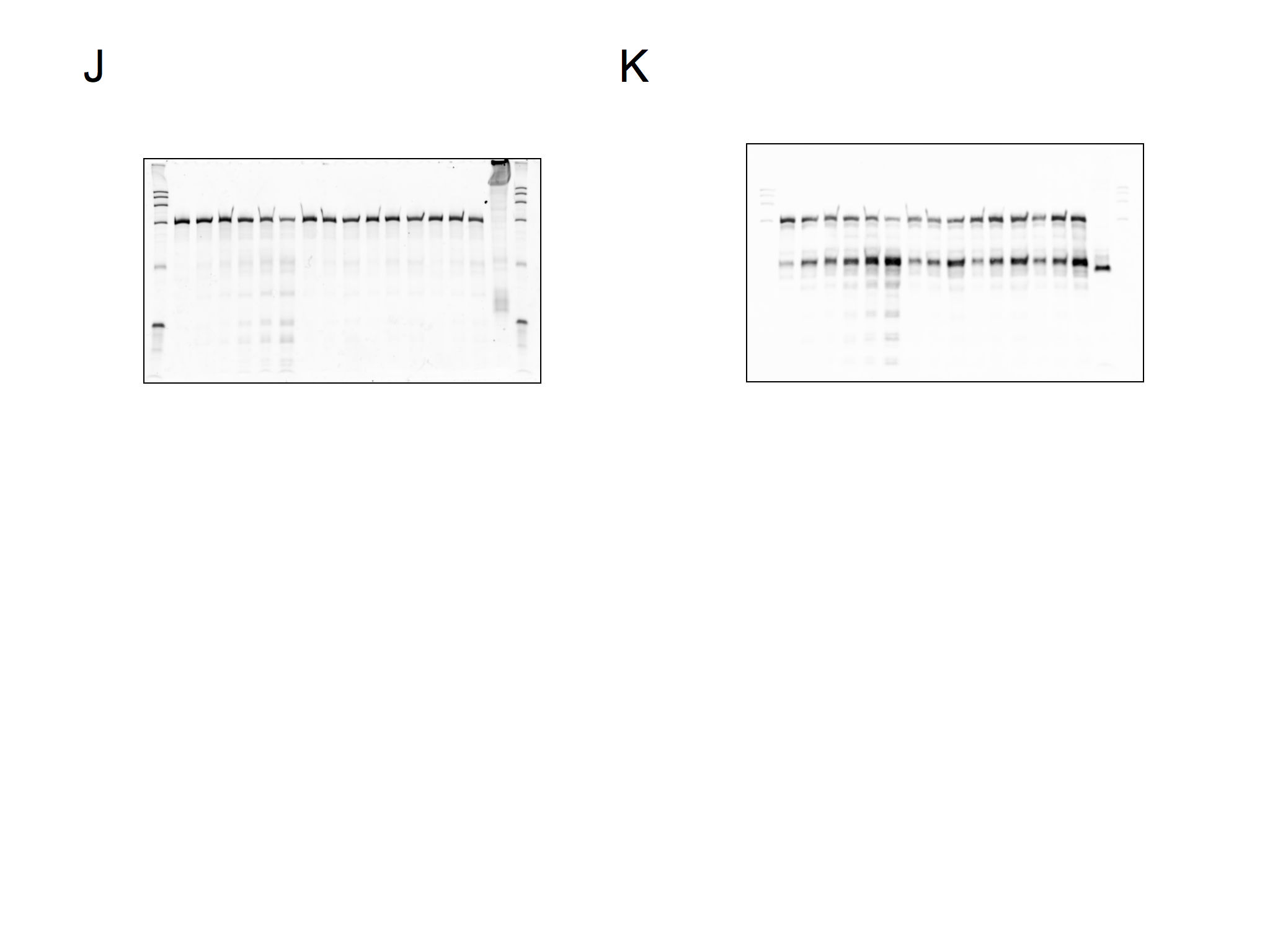


Fig. S6. Raw data of Figures

(A) and (B) Raw data of Fig. 2A. (A) The gel image of SYBR II staining before Northern blotting analysis of Fig. 2A and (B) raw data of Fig. 2A.

(C) and (D) Raw data of Fig. 2C. (C) The gel image of SYBR II staining before Northern blotting analysis of Fig. 2C and (D) raw data of Fig. 2C.

(E) Raw data of Fig. 3.

(F) and (G) Raw data of Fig. 4A. (F) The gel image of SYBR II staining before Northern blotting analysis of Fig. 4A and (G) raw data of Fig. 4A.

(H) and (I) Raw data of Fig. 4C. (H) The gel image of SYBR II staining before Northern blotting analysis of Fig. 4C and (I) raw data of Fig. 4C.

(J) and (K) Raw data of Fig. 5C. (J) The gel image of SYBR II staining before Northern blotting analysis of Fig. 5C and (K) raw data of Fig. 5C.

Table S1. Comparison of the lengths of the rRNAs in KUW1 and *Δfau-1* cells.

|  |  | KUW1 (N = 6) | | *Δfau-1* (N = 6) | |  |
| --- | --- | --- | --- | --- | --- | --- |
|  | OD660 | Average (nt) | S.D. | Average (nt) | S.D. | *p* value |
| 5S rRNA | 0.05 | 91.5 | 0.2 | 90.8 | 0.3 | 0.0250^*^ |
|  | 0.1 | 91.8 | 0.4 | 91.7 | 0.3 | 0.6108 |
|  | 0.2 | 91.0 | 0.5 | 91.8 | 0.7 | 0.4743 |
| 16S rRNA | 0.05 | 1607.0 | 8.7 | 1620.3 | 8.6 | 0.0240^*^ |
|  | 0.1 | 1578.2 | 9.4 | 1579.8 | 7.1 | 0.7988 |
|  | 0.2 | 1577.8 | 9.3 | 1566.0 | 8.6 | 0.0253^*^ |
| 23S rRNA | 0.05 | 3380.2 | 26.9 | 3436.5 | 27.5 | 0.0066^**^ |
|  | 0.1 | 3271.0 | 39.4 | 3229.0 | 20.8 | 0.0835 |
|  | 0.2 | 3204.7 | 27.8 | 3158.8 | 19.7 | 0.0083^**^ |

Statistically significant differences (Student’s two-tailed t-test): **p* ≤ 0.05, ***p* ≤ 0.01.

Table S2. Sequences of the PCR primers used in this study.

| Name | Sequence (5’→3’) |
| --- | --- |
| S0009 | 5’-AAAGATCCTGCAGGTGCGTCA-3’ |
| S0010 | 5’-CGCCGTACCACTACCAACTC-3’ |
| S0011 | 5’-ATGGCCGCCGTACCACTACC-3’ |
| T0005 | 5’-GGAATTCCATATGTCTACAGACACAAGGCCTACAGTC-3’ |
| T0006 | 5’-CCGCTCGAGAACCCTCTCAAATACCTCCTGCGTTATCC-3’ |
| P0032 | 5’-AGTGTTGATCTTGGAGAGTGG-3’ |
| P0031 | 5’-CACGTGTCTCTGAACTCCTAT-3’ |
| P0034 | 5’-AACACGTTAAGGGATGAACTA-3’ |
| P0033 | 5’-TAGTCCCAGGATTCTCAACCT-3’ |
| P0038 | 5’-GAGAAGTTCTCAGCTCCAGCAGAGATA-3’ |
| P0039 | 5’-CCAATCCTTGTAAGCGGCAGCAGT-3’ |
| P0044 | 5’-TAATACGACTCACTATAGTCCAAATACTAGCTACAACCGT-3’ |
| P0007 | 5’-GATAGTGGCCGGCGGCGTCCCCG-3’ |
| T0009 | 5’-TAATACGACTCACTATAGCTCCTCCATCGTGACTCGCTTG-3’ |
| T0031 | 5’-GTGGCCGGCGGCGTTCCCGGTTTCC-3’ |
| FAU_A5 | 5’-TCCTCTGGAGTGAGCTCGTTTTTCGCCAGA-3’ |
| FAU_A3 | 5’-TTTCAGGATAACGGAGAGGGGAATAGAGGAC-3’ |
| FAU_B5 | 5’-CTAATTCTGACTGTAGGCCTTGTGTCTGTA-3’ |
| FAU_B3 | 5’-GGCAGGGTCTTGATTTCCAGCTTTTCAGCCTC -3’ |
| FAU_C5 | 5’-ACGCCGGTTATCCTTTTGTAAACCTCCGGC-3’ |
| FAU_C3 | 5’-AAGCGGCCTGATAGCTGAACTGAGCAGACA-3’ |
| N0009 | 5’-ATTCCGGTTGATCCTGCCGGAGGC |
| N0010 | 5’-TGCCCCTTTCGGCCTGAGGACC |
| N0011 | 5’-GCCCGGTGGCAACTAAGCCGCCTG |
| N0012 | 5’-TTCCTGCGGGTACTAAGATGTTTC |
